# Soluble Salts Quantitative Characterization and Thermodynamic Modeling on Roman Bricks to Assess the Origin of Their Formation

**DOI:** 10.3390/molecules26102866

**Published:** 2021-05-12

**Authors:** Claudia Scatigno, Nagore Prieto-Taboada, Giulia Festa, Juan Manuel Madariaga

**Affiliations:** 1 CREF-Museo Storico della Fisica e Centro Studi e Ricerche “Enrico Fermi”, Via Panisperna 89 a, c/o Piazza del Viminale 1, 00189 Rome, Italy; giulia.festa@cref.it; 2 Department of Applied Chemistry, Faculty of Chemistry, UPV-EHU, P° Manuel Lardizabal, 3, 20018 Donostia, Spain; nagore.prieto@ehu.eus; 3 Department of Analytical Chemistry, Faculty of Science and Technology, UPV-EHU, 48080 Bilbao, Spain; juanmanuel.madariaga@ehu.eus; 4 Unesco Chair of Cultural Landscapes and Heritage, UPV-EHU, 01006 Vitoria-Gasteiz, Spain

**Keywords:** efflorescence and sub-efflorescence quantitative characterization, Roman bricks, ion chromatography (IC), salts decay origin, thermodynamic modelling, ECOS-RUNSALTS, Medusa-Hydra

## Abstract

The environmental weathering and the formation of efflorescences on the brick walls are studied at the “Casa di Diana” Mithraeum at Ostia Antica archaeological site. Previous studies on subsoil, bedrock, hydrological systems and environmental conditions, and new ion chromatography analysis combined with ECOS-RUNSALT and Medusa-Hydra thermodynamic modelling software, had allowed us to identify the subsoil contamination related to soluble salts. The atmospheric acidic gases, CO_2_ and SO_2_, are determined as the main salt weathering species. A dry deposition after a subsequent hydration action from the shallow freshwater aquifer that reaches up to 1 m on the walls is identified as the mechanism of salt formation. An evaluation of potential sources such as the nearby Fiumicino airport, CO_2_-rich gases inputs from fumaroles and CO_2_ inputs was also debated. The risk level of contamination the surfaces of the materials should be considered mildly/very polluted with a medium/high risk of hygroscopic moisture due to the high concentration of sulphates.

## 1. Introduction

Historic buildings have a complex relationship with the environment they are set in, being constantly influenced by external factors [1]. In the context of archaeological sites, the buildings kept inside, fully, or partially protected by roofs (original or not), undergo several decay processes due to their exposure to aggressive environmental conditions [2,3,4,5]. Several issues can influence the durability and preservation, speeding up natural and progressive decline. Some of them are: (1) the inner geometry, (2) the materials of construction and (3) the location of the building [6].

The response of the building materials to these external solicitations (environmental stressors) results in degradation of the materials [7]. Amongst them, soluble salts represent one of the main conservation problems, because their presence within the capillary network causes the dissolution of building materials, material loss and cracks [8]. Specifically, the growth of salt crystals within the pores of a stone could generate stress (internal tensions), either by their crystallization or by changes in volume (expansion of crystals) according to the number of hydration water molecules. The availability of water, carrying dissolved ions or promoting their dissolution from the materials, is a key step in the crystallization of salts [9,10,11]. Indeed, in addition to the resulting aesthetical problems, soluble salts also produce a considerable decay when salts precipitate beneath the material as sub-efflorescence [12]. However, not all salts are equally harmful and for this reason, the identification of their nature is crucial [13]. In fact, the possibility of dehydration–hydration processes plays a critical role in the deterioration mechanisms, as in the case of sodium sulphates, which are thoroughly studied [14,15,16]. Furthermore, the nature of the salts is not the only important aspect, as their concentration is also a crucial factor to evaluate the conservation state of a building [17].

Some studies have analyzed the action of sea salt aerosols on the construction materials of historical monuments [18,19,20,21]. In coastal regions the atmosphere could be enriched with particles that are naturally generated by the action of wind on the water surface [22]. These particles make up the sea spray, which introduces ionic species into the atmosphere, principally chlorides and sulphates [23,24].

To identify the nature of the salts and to quantify them, the most useful technique is ion chromatography (IC) after the extraction of soluble salts from the brick walls. In this sense, this technique is widely used, even being regulated by a standard protocol [25]. Moreover, this procedure is not only reliable for determining the level of soluble salts, but also to assess the effectiveness of salt removal methods in cultural heritage materials [26,27,28,29,30,31,32,33,34,35].

Besides the salts’ typology (solubility, hygroscopy or hydration level) and the characteristics of the material (mainly its porosity), other factors that regulate crystals’ formation are the thermo-hygrometric conditions (frequency variation) [36]. Indeed, apart from the walls directly exposed to outdoors (where the pollutants also are regulated by long-range transport), the processes of salt formation are linked to the micro-environmental conditions. Specific ranges of relative humidity (RH) and temperature (T) govern the evaporation and condensation phenomena, producing efflorescences [37,38]. Phase transitions occur at a specific RH for each salt, at a given temperature and pressure, called equilibrium relative humidity [39]. In this sense, knowledge about the salt behavior, and if possible, the changes in T and RH conditions, is crucial [40]. Moreover, single salts are rarely found in nature. In practice, buildings are contaminated by salt mixtures, which present a totally different behavior. Therefore, the assessment of the critical environmental conditions of salt laden porous material and hence, potential risks of salt damage, requires knowledge of the thermodynamics of the relevant chemical equilibria [40]. In this sense, thermodynamic modelling represents an important tool to understand the salts’ formation and, subsequently, the ways to avoid it, safeguarding historical buildings.

The literature on the simulation of salt damage on porous materials is well disseminated [41,42,43,44], as is the use of particular thermodynamic software packages [45,46,47]. In this sense, there are two interesting thermodynamics modelling programs, ECOS-RUNSALT [48,49,50] Medusa-Hydra [51,52], whose usefulness in cultural heritage research is widely demonstrated. The first program utilizes a thermodynamic model to predict which solid minerals (salts) exist in equilibrium taking into account only a certain thermo-hygrometric range [53]. It does not allow maximum values of relative humidity (RH_max_) exceeding 98% (an important limit in the case of gypsum). Moreover, the program has several restrictions correlated to the input of data, as anions like carbonates and cations like ammonium and barium are not considered by it. Furthermore, on some occasions, the prediction of gypsum crystallization might cause problems, since the program is unable to calculate the crystallization of other salts in its presence, and gypsum must therefore be removed from the system. Due to these restrictions, the model can only applied only to some limited cases (listed successively), because of the abundant presence of calcium in many materials under study. The second, a freeware chemical equilibrium software developed by the KTH School of Chemical Science and Engineering (Stockholm, Sweden) [53], is widely used for theoretical analysis of thermodynamic feasibility and the existence of metal speciation in aqueous solutions analyzed through speciation diagrams, proving powerful and comprehensive visual summaries of the solubility relations in aqueous process systems. Indeed, it can show all the possible complex species that could exist in the wide range of pH values. The theory is based on how in the typical pH range used the dominant species form stable complexes.

On the other hand, the second program allows one to predict the chemical equilibria in dissolution with less limitations than ECOS-RUNSALT, but considers a fixed T value of 25 °C. Despite the availability of these environmental tools, the processes and pathways of salt damage are still incompletely understood [54,55].

Here, taking advantage of the knowledge of the site (subsoil, bedrock and environmental conditions) obtained [4,45,56,57,58,59,60,61,62,63,64,65], a methodology that combines both the thermodynamics modelling methods is performed. Moreover, for the first time these software packages are tested together on a “hypogeum environment” case, characterized by extreme environmental conditions. Understanding one of the main important problems in decay and preservation of cultural heritage sites and buildings, is essential to assess an adequate conservation plan for a cultural site.

In detail, a methodology based on a quantitative characterization (IC) combined with two thermodynamic models (ECOS-RUNSALTS and Hydra-Medusa) were applied on the building walls (red and yellow bricks) of a complex site at Ostia Antica (Italy), with the aim to study on the origin and the mechanism of salt formation, pinpointing the source(s) of the salts that at the first sight are multiple.

## 2. Materials and Methods

### 2.1. The Building under Study, the State of Conservation and the Environmental Scenario

**Figure 1:**
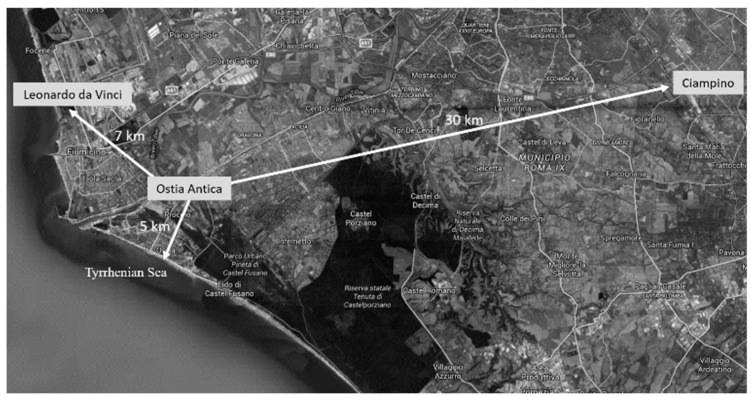
Ostia Antica map which allows one to observe the distances and orientation in relation to the two airports (Leonardo da Vinci on the left and Ciampino on the right) and the Tyrrhenian Sea.

The cultural site of Ostia Antica (Rome, Italy) is, together with Pompeii and Herculaneum, one of the best-known archaeological examples of Roman houses found in Italy. Ostia Antica was Rome’s first colonia and played an important role as a port to supply the city of Rome. This cultural site is near to the Tyrrhenian sea (at least around 3 km south), in the proximity of the Ciampino (around 30 km east) and Leonardo da Vinci (commonly known as Fiumicino, around 7 km northwest) airports and very close to the urban city center (around 5 km) (Figure 1).

**Figure 2:**
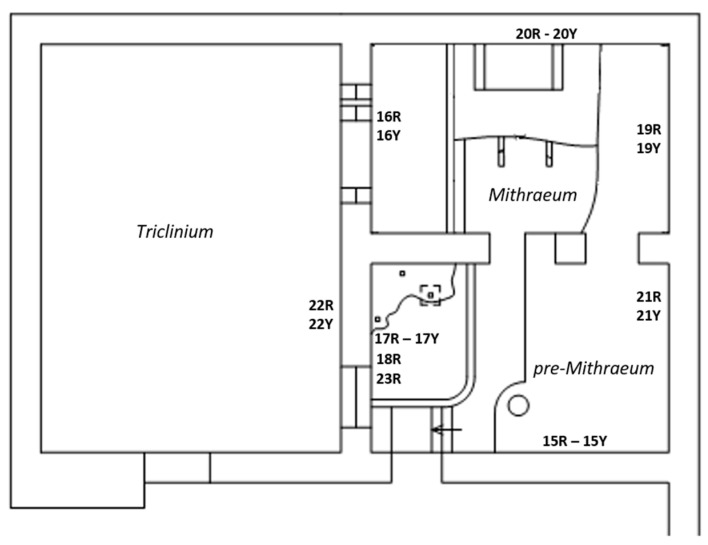
Plan of the building and measuring points.

Amongst the Roman masonry examples present in Ostia Antica, we focused on a building, called “Casa di Diana” or “Caseggiato di Diana”, dated 150 CE and particularly on the last two inter-communicating rooms, located on the northern side of the building, the “*Mithraeum*” and “*pre-Mithraeum*” (Figure 2).

**Figure 3:**
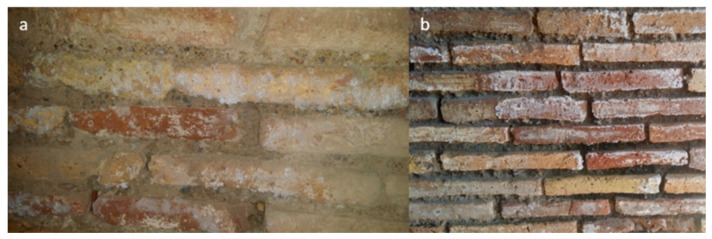
Examples of the soluble salts’ formation on walls. (**a**) The mattifying veil and the efflorescences on south wall of the *pre-Mithraeum* (indoors). (**b**) The opacifying and the efflorescences on south wall of the *Triclinium* (outdoors).

This is a place that was dedicated to the cult of the Persian god Mithra during Roman times. A roofless room named *Triclinium*, neighboring with the two rooms, was also considered. The building was affected by efflorescences year-round (Figure 3) and rising damp due to the presence of two aquifers (freshwater and salt water) at shallow and deep depth (2.5 and 8–10 m) [42,57,58]. These water masses are precursors and responsible of the high RH values (close to saturation), recorded in the lowest area of the building (0–1.1 m) during indoor microclimatic study campaigns [42,56]. Although it is believed that a possible impermeable stratum (natural membrane of sand alluvial deposits) separates the two “water pockets”, the influence of the deepest salt aquifer [57], through the rising damp mechanism, could be involved in the salt formation mechanism due to the fact the foundation wall reaches the freshwater–saltwater interface.

Furthermore, the environmental surveys, which define this place as a “*hypogeum environment*” (an underground room simulating a cave) indicate that the building is also affected by low air flows, facilitating evaporation and condensation phenomena near the walls [64]. Relative insolation from the outdoors, besides the presence of several openings (especially of one window of considerable dimensions sited on the east side) and lower recorded air velocity values [64] (inside and outside-*Triclinium* Figure 2) also suggest a non long-range transport of eventual pollutants. In this sense, studies on air quality established very high concentrations of CO_2_ (of biological and anthropogenic input) throughout the whole day (night cyclic bio-emissions and day emissions in correspondence with the high turnout) [61] because of the inadequate air inlet flow. Values about two-fold higher than those monitored outside were recorded, so the CO_2_ indoor inputs must be considered [59].

Finally, regarding the salt origin, especially of Na^+^ and Cl^−^, studies conducted on the water levels of the Tiber delta, by sampling 120 wells located in the surroundings, also revealed the presence of saltwater intrusion and, therefore, widespread salt contamination from the subsoil due to the relative closeness of the Tyrrhenian Sea (only 3 km away). Moreover, the salt marshes were exploited until 1895 [8]. This last issue probably explains the contribution of salts found in well water samples in previous geochemical analyses [57].

However, there is another possible source to be taken into consideration: the rainfall (mixed with atmospheric acid gases and/or marine aerosol) that could be also the responsible of the salt formation by infiltration from the roof and the *Triclinium* room (Figure 2) into the brick detachments or even through the openings (i.e., the window of the *pre-Mithraeum* room).

Thus, the diversity of sources that could be affecting the building, from the bottom upwards, is important and clarification of the proper source that promotes the formation of the soluble salts is essential in the design of any conservation plan for the building.

### 2.2. Sampling Procedure

The sampling methodology was designed to evaluate the factors that could have an influence on the salt formation. In this sense, two different typologies of bricks (red and yellow, identified as R and Y, respectively—see Table 1) were sampled on different walls (building orientation), because they are subjected to different environmental conditions [60]. To minimize the micro-sampling and considering that the rising damp reaches up to 1 m [4], a value confirmed also by moisture measurements conducted directly on wall-building materials along a vertical profile [42], it was possible to assure the significance of the collected data, selecting specific areas. Although in general the whole building is affected by rising damp, and typical vertical profiles were observed [61], in this case, no correlation with the total content of soluble salts (up to 3 m) was found [4].

In this sense, it was possible to implement a reasoned non-probabilistic sampling design (Table 1), with the selection of only 18 solid samples, to identify and to characterize the observed soluble salts. Specifically, 16 bricks were sampled at 1.03/0.65 m also considering the orientation and the typology of bricks (red and yellow).

Two other samples of salt efflorescences were also taken from two walls with different orientation and found in different rooms (24S_1_ and 25S_2_). Furthermore, one water sample was also obtained (W_w_) from the well, to characterize the type of water) and, indirectly, also from the (communicated) freshwater aquifer. Finally, one rainwater sample (T_1_) was collected during a geochemical study conducted in November 2015 [57]. Despite the fact sampling of the deepest water mass was not possible, it was classified in previous studies as marine water [57,58].

Thus, the diversity of sources that could be affecting the building, from the bottom upwards, is important and the clarification of the source that promotes the formation of the soluble salts is essential to the conservation plan of the building.

### 2.3. Analytical Procedure

Regarding the analytical method, all the samples were crushed in an agate mortar and dried in a drying cabinet (60 °C) until a constant weight was obtained (24 h). The soluble salts were extracted by an ultrasound-assisted procedure with water (100 mg of sample in 100 mL of MilliQ water), following an optimized methodology based on the European standards [66]. This pre-treatment was replicated four times for each analyzed sample. After the extraction, the obtained solutions were characterized by an ICS 2500 ion chromatograph (Dionex, Sunnyvale, CA, USA) equipped with an ED50 suppressed conductivity detector. An IonPac AS23 (4 × 250 mm) column and IonPac AG23 (4 × 50 mm) pre-column from Dionex were used for the separation of anions. The quantification of cations was conducted using an IonPac CS12A (4 × 250 mm) column and IonPac CG-12A (4 × 50 mm) pre-column from Dionex. The chromatographic conditions used in the anion quantification were 5 mM Na_2_CO_3_/0.8 mM NaHCO_3_, 25 mA and 1 mL/min as mobile phase, suppression current and flow, respectively. In the case of cations, 20 mM CH_4_SO_3_ as mobile phase, 59 mA of suppression current and 1 mL/min flow were used. Prior to the analysis, the samples were passed through a 0.45 μm nylon syringe filters and brought to a final volume.

To estimate the carbonate concentration, the pH values of the extracted soluble salts were measured. Thus, the pH measurements were conducted with SOILSTIK pH meter (Spectrum Technologies, Inc., Aurora, IL, USA) and were replicated three times. Data was treated with OriginPro version 8.5.1 (OriginLab©, Northampton, MA, USA).

To deepen the saline intrusion and the possibility of the attack on the wall-building materials by rising damp, a sound velocity study was conducted at the beginning of September 2016 (per 1 day) using a probe (Monitor SVP v. 2c, Valeport, UK) placed in the well. The aim was to validate the effect of the shallow freshwater aquifer according to the sound velocity reference values. Sound velocity, P and T were recorded during the first 5 min of the measurement (sampling time of 1 min at 1 Hz, disregarding the decreasing and climbing measurements), giving the corresponding values. The accuracy of the sound velocity, T and RH measurements are ±0.02 m/s, ±0.01 °C, ±0.1%, respectively.

Regarding the environmental monitoring, the thermo-hygrometric data, derived from a wide microclimatic campaign conduced from 2012 to 2015 [42] were inserted in the computer program Environmental Control of Salts (ECOS)-RUNSALT (version 1.9) [67,68,69], according to the allowed range configured, to predict the soluble salt formation under the micro-environmental conditions (annual average). The latter was used in combination with another thermodynamic software program, Medusa-Hydra (v. 2010). Specifically, the model was used to assess if sulphate salts (threatening for stone materials) can mainly be formed because of the attack of SO_2_ on the calcite materials. In this sense, calcium carbonate was introduced in the input and the accumulative effects of the SO_2_ impact were modelled with the gradual addition of SO_2_ (g) (from 0 to 500 mg/L^−1^ or ppm). If SO_2_ impacted (an accumulated attack equivalent to a concentration of 50 mM was considered) the system in question (calcite immersed in an oxidative environment, equivalent to 75 mM of oxygen/ozone), the calcite would be gradually transformed. The thermodynamic prediction by ECOS-RUNSALTS was also supported by parallel ongoing studies using the Raman spectroscopy, micro-X-ray fluorescence, and X-ray diffraction techniques performed on these Roman bricks [59] where a sulphate and carbonate attack on bricks was established especially for the yellow ones [59].

## 3. Results and Discussion

Thanks to the quantitative analysis, F^−^, ClO_2_^−^, Cl^−^, NO_2_^−^, NO_3_^−^, PO_4_^3−^ and SO_4_^2−^ anions and Na^+^, K^+^, Mg^2+^ and Ca^2+^ cations, were quantified. The soluble salt results are shown in Table 2.

Because of the nature of the mobile phase used, it was not possible to analyze the concentration of any dissolved bicarbonate. This was then determined theoretically through the completion of the mass balance and assuming electroneutrality in the liquid extracts after performing the soluble salt tests (1) [70]:

(1) ∑(valence cation conc.)−∑(valence anion conc.)=bicarbonate concentraion 

**Figure 4:**
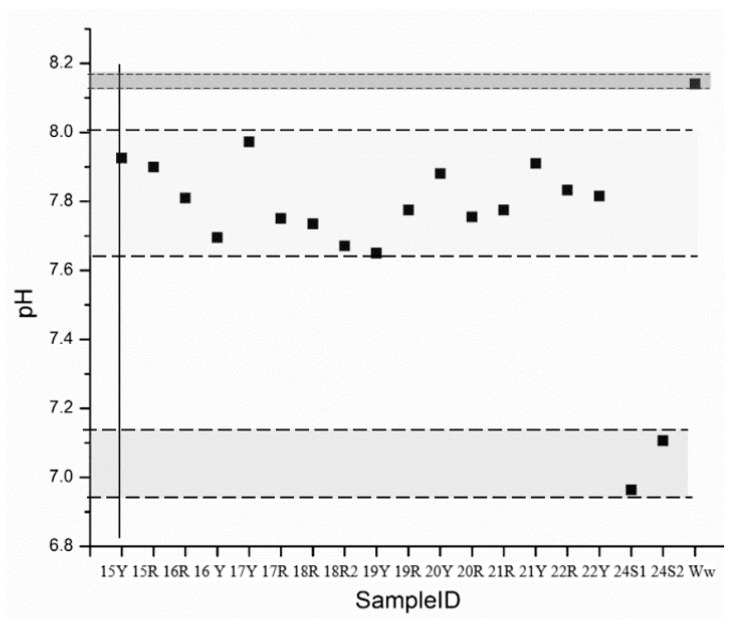
pH measurement scatterplot.

To estimate the presence of carbonate or bicarbonate, pH measurements were required. All the bricks samples presented a pH below 8, as can be seen in Figure 4, thus, bicarbonate was estimated [70]. In the case of water and salts samples, bicarbonate was also assumed.

**Figure 5:**
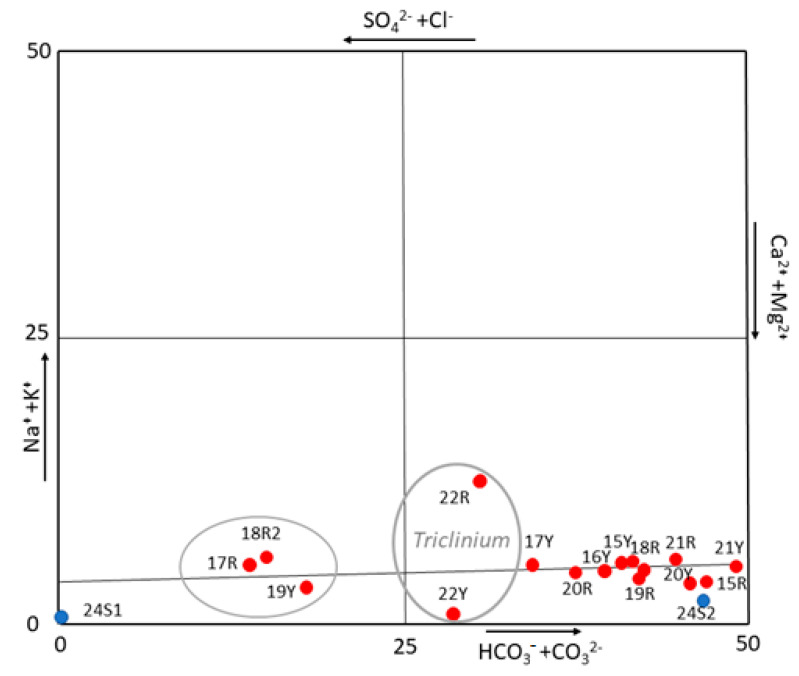
Anion and cations diagram (visualization and classification of hydro-chemical data) which excludes the marine water as origin of salts.

To better understand the origin of these salts, a correlation diagram, normally used in hydrogeochemical studies [70], was plotted using the results shown in Table 2 (Figure 5). In fact, the diagram visualizes and classifies the hydrogeochemical facies (one or more) and the dominant one(s) among them, providing a source classification [43,60]. Considering that the water extraction decreases the ion concentration in the solution but not the ionic ratio, the use of this diagram could help to identify the type of water that mobilizes the salts. Thus, the diagram reveals carbonate species such as Ca^2+^ and Mg^2+^, that is, of carbonate origin. Nevertheless, extracting more information about the origin of these carbonates was difficult because the results could indicate freshwater but also, the dissolution of the building materials by the action of the rainwater or dissolution of carbonate materials (bedrock). However, the quantitative results and this diagram excluded the action the mass of water of the known deep (salt water) aquifer as responsible for the rising damp because of the absence of high concentrations of anions such as Cl^−^, Na^+^ or K^+^.

Moreover, the result conducted on well water by the sound velocity probe, confirmed again that the measured water was fresh water, not salt water [56]. In fact, the brackish water was characterized with values around 1485 m/s until 1495, while the surface Tyrrhenian Sea waters were 1509 and 1540 m/s in winter and summertime, respectively (values generally influenced by only the temperature parameter thermocline). The obtained results were compatible with the theory of Cutnell [53].

**Figure 6:**
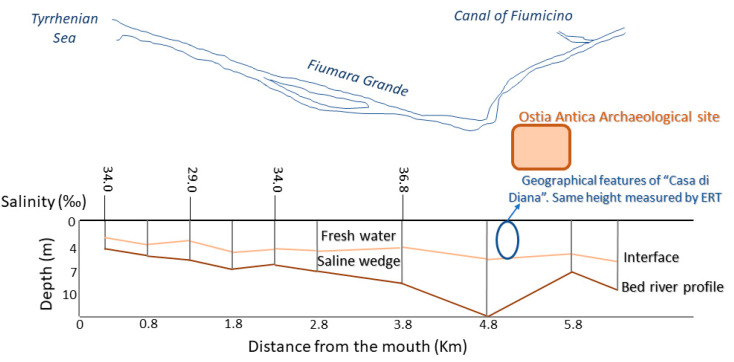
Schematic cross-section of the saline intrusion and the bathymetry along the median axis of the “Canale della Fiumara Grande”, from Capo Due Rami to the river mouth [71]. The cross and the circle indicate the Ostia Antica site and “Casa di Diana” building (with the ERT measurements), respectively.

An ulterior evidence of the not-existent influence of the saline water aquifer on the freshwater aquifer was derived by hydrological and chemical investigations conducted in the surroundings of the site under study in 2007 [71]. The researchers then studied the water levels of the Tiber delta (sedimentary bodies’ reconstruction) by sampling on 120 wells located on the surroundings. These observations revealed the presence of salt-water intrusion and, therefore, a widespread salt contamination of the Tiber delta that constitutes a very high risk to water management practices. In Figure 6 it is possible to observe that the saline intrusion in the “Canale della Fiumara Grande” that overreaches from the mouth and goes inwards with salinity high values (37 g/L). In the same vertical section, two sub-layers of freshwater and salt water with a sub-horizontal trend (interface) were identified, as in the case of “Casa di Diana” (in correspondence with salinity values of about 38 g/L).

Furthermore, the presence of significant hollows along the longitudinal bed profile, promotes the saline intrusion stagnation, which lasts for long times, despite the variability of the river system and the marine weather conditions. For all these reasons, the study of 2007 was in accordance with the electrical resistivity tomography (ERT) results obtained [57], as can be seen in the Figure 6, with the identification in the “Casa di Diana” (circle) at 5 m of depth of a section attributable to “river fresh water” and below it, a salt-water intrusion plume. In the mentioned work, and the despite of the variability of the river system according to the weather conditions, the saline intrusion is excluded (but not underestimated, assuming groundwater vulnerability). Thus, the interface line between salt and freshwater shown in the Figure 6, indicates the coexistence of two distinct masses of water (salt and fresh water) separated by a natural membrane [57].

Taking all these results into account, one of the three possible salt sources (salts carried by rising damp coming from the saline aquifer or saline intrusion from the sea) could be discarded, even if circulating minerals and salts (derived from the exploitation of the salt marshes) in the subsoil should be involved.

To find possible relationships among the concentrations collected in Table 3, a correlation analysis was performed using only the data from bricks (16 samples). The results are summarized in Table 3 showing that the correlation between Ca^+2^/SO_4_^2−^ was likely (r = 0.96).

Thus, gypsum formation had a high possibility of also occurring in the brick samples. That is, a source of sulphates is affecting to the whole volume of *Mithraeum* because even the bricks at the highest level in the walls are partially sulphated in their surfaces.

Concerning the different salts that can be formed, the quantitative and correlation analyses identified gypsum and calcite as the main salts. For this, we focused the attention on these types of salts.

To confirm their main presence, a first thermodynamic modelling was carried out using the ECOS-RUNSALT software, because it allows one to introduce the RH as an input parameter, a very important factor in this specific indoor environment where although the average RH value is 95.6%, in the lowest strata (0–1.1 m) the recorded RH values are very close to saturation (96–99%) [42]. Moreover, a previous monitoring campaign had revealed that the T is stable (T_my_ 16 °C) throughout the entire year and the daily T variations are not significant [42].

Due to the restrictions described in the experimental section the ECOS-RUNSALT model could be applied only to some of the described cases (15R, 16R, 18R, 21R, 21Y), due to the abundant presence of calcium in these samples.

Leaving these considerations aside, regarding the impossibility of introducing barium and ammonium, it was not a problem due to the undetectable concentrations of both cations. This fact does not break the system electroneutrality because they can be considerable as negligible compared with the rest of ions. On the other hand, as it is possible to include carbonate in the model, the electroneutrality of the samples breaks down. Observing that the values of concentration of both ions were quite similar, calcium and carbonate total concentration values were removed from the system to maintain the electroneutrality.

**Figure 7:**
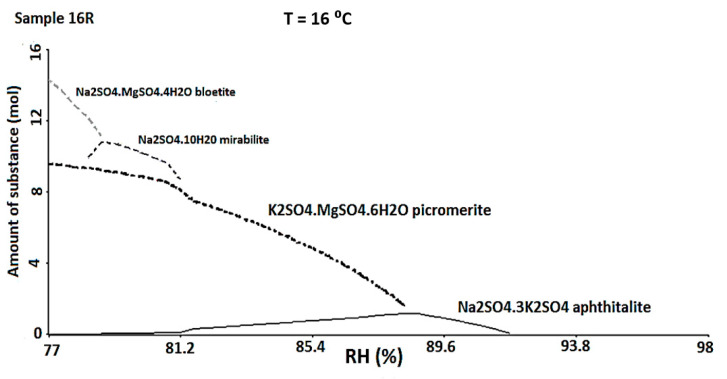
ECOS-RUNSALT output (16 °C, RH 98–77%) for the sample 16R in which is possible to observe the formation of different sulphates.

The ECOS-RUNSALT simulation was carried out considering a T_my_ of 16 °C, a RH_max_ of 98%, a RH_min_ of 77% and the ionic concentrations obtained in the quantitative analysis (Table 2—final input data). Within these thermohygrometric ranges, the program predicted the formation of specific minerals that indicated again a strong attack deriving from SO_4_^2−^. In particular, the 16R and 18R red bricks presented the same phases (Figure 7): aphthitalite ((K,Na)_3_Na(SO_4_)_2_), picromerite (K_2_Mg(SO_4_)_2_·6(H_2_O)), mirabilite (Na_2_SO_4_·10H_2_O), and bloedite (Na_2_Mg(SO_4_)_2_·4H_2_O). In the 21R and 21Y samples aphthitalite and picromerite were suggested to be present by the simulation. Finally, picromerite was the only phase predicted in 15R. It is important to underline that at these RH values, the only phases that prevail are mixtures derived from sulphates. Usually, the composition of marine aerosols is dominated by halite (NaCl), even if the sea salt contains other species that form a complex mixture [72]. If the marine aerosols were affecting the external building walls and penetrating by capillarity or through openings inside the inner building walls, according to the recorded micro-environmental, any halite should be solubilized. It is interesting to note that the samples are collected also up the 1 m height (where de RH does not exceed 98%). During the simulation, several tests, according to the samples collected, where carried out, changing the relative RH, but halite was never predicted. The salt mixtures that the model predicts are always the same and seem differ considerably from the composition of sea salts.

It is equally true that the “fractionated infiltration”, or “alternative salts’ contamination” pathways, generates a continuous deposition and removal of salt efflorescence that differed considerably from the composition of sea salt [15].

**Figure 8:**
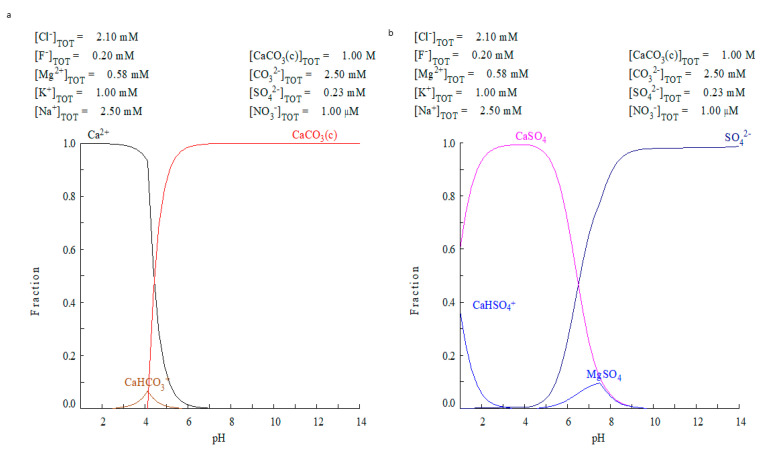
Medusa-Hydra output for rainwater (T_1_) interaction with calcium carbonate. (**a**) Calcium species distribution vs. pH and (**b**) sulphate species vs. pH. In neither case is the observed salt formation explained.

Considering the limitations of ECOS-RUNSALT, a second simulation strategy was performed using the Medusa-Hydra software on the water solution (outer tank—rainwater and inside the well—freshwater). In a first attempt, the ionic concentrations of rainwaters were studied (Figure 8a). In a second attempt, the ionic concentration of the freshwater of the shallow aquifer (26W), was used as input data for the program (Figure 8b). Both types of water presented lower concentrations of dissolved ions (max. 0.029%) at pH values around 8. Figure 8 shows the distribution diagram of calcium (Figure 8a) and sulphate (Figure 8b) considering that rainwater interacts with a calcite substrate. As seen, the calcite starts to be dissolved at pH < 7, but the rainwater has a pH~8, not acid enough to attack calcite (Figure 8a).

Furthermore, considering the sulphate species, the rainwater cannot explain the formation of any solid sulphates like gypsum, aphthitalite, picromerite, mirabilite or bloedite (Figure 8b). On the other hand, for the simulation using the concentrations of the well water sample (W_w_), the results were the same: any solid sulphate species were formed from the calcite substrate. We observe only the presence of sulphates related to the salts circulating in the subsoil (CaSO_4_ and MgSO_4_). Thus, the freshwater of the aquifer at shallow depth and rainwaters, both connected with the building (hydrologic setting of the house) are not enough to degrade the calcite and promote the formation of the predicted solid sulphate species.

Taking all these facts into account, the source of sulphate should be linked to atmospheric SO_2_ attack. This attack could occur by two ways, namely dry deposition, and wet deposition. When the latter occurs a high concentration of sulphates and low pH of the rainwater is expected, but in this case, this fact was not observed, suggesting a dry deposition mechanism. Moreover, another source of the sulphate salts could be because of the rising damp coming from the subsoil (the first 2.5 m) due to the exploitation of the salt marshes, which although they have low concentration of salts, the cumulative supply of these salts circulating in the subsoil by rising damp in the building walls should be considered. Anyway, the low pH requirement indicates more probably the action of atmospheric acid gases, at least to promote the dissolution of the original calcite (Figure 8), otherwise the degradation of the calcite was not occurring only observing the accumulation of salts in the materials. For that reason, the interaction with atmospheric acid gases is very plausible.

### Possible Mechanisms

Thanks to all these observations it was possible to propose a mechanism for the formation of the salts: (1) a hydration process of the original oxides by H_2_O to form reactive hydroxides. (2) Attack by indoor CO_2_ inputs on wet bricks to form carbonates and net acidic water. (3) Dissolution of the carbonates and SO_2_ attack with dry deposition to form sulphates. Considering the proposed mechanism, the porosity of the materials and the salts’ mobility, the formation of sub-efflorescences is possible, but the phenomena is expected to be more intense on the interface with the atmosphere (efflorescences), as is the case studied. Nonetheless, the key step is the hydration of the original compounds, because without this, the attack of the acid gases by dry deposition is unlikely.

Once the mechanism is understood and the several pollution sources evaluated it is necessary to narrow down their source to try to contain it. It is noticeable that Ostia Antica is located about 5 km from the seacoast in the south direction, so marine aerosols are expected to play a considerable role. It is also known that the wind speed is the key factor to determine the production rate of many physical processes over the ocean surface that can generate sea salt aerosols. Studies on the relationship between the salinity and distance from the coast reveal an exponential decrease of the marine aerosols with the increase of the distance from the coast. In fact, it was observed that five kilometers seem to be enough to minimize the contribution of the marine aerosol [73,74]. Moreover, the air turbulence in the interior of building is zero, also considering the size of the openings (approximately 0.04 m/s). This means that, on the one hand, the condensation phenomena are encouraged (corners of the building and up to 1 m from the ground), while on the other hand, the low wind speed makes more difficult the pollution transport inside the building (through the main openings). Furthermore, it is known that the wind direction changes, but the prevailing winds come from the northwest, that is the direction of the Leonardo da Vinci airport (around 7 km away from the site). Moreover, according to the results listed in Table 2, no appreciable difference was found between the outer bricks and the inner ones. Additionally, according to the salt presence study conducted in 2014, the total content of soluble salts were more than double on the inner wall compared to the outer one, sulphate and chloride being more abundant in the inner wall [45].

Another aspect to take into consideration is the CO_2_ input (another important gaseous acid). Studies on the prevalent air quality show that the concentrations of CO_2_ are relatively high in comparison to the suggested limits and guidelines defined by law [75,76,77,78,79,80]. The inputs deriving from the biological environment and from inadequate air movement cause poor air quality and possibly accelerate acidification, due to the combined high RH values close to saturation [42] of the stone materials. Remaining on the topic of emissions, one aspect to consider is the surface manifestation of CO_2_-rich gases in the area surrounding the archaeological site (fumaroles). In the coastal area of Fiumicino, not far away, leaks of natural gases (putting at risk the population) have manifested themselves for a long while [81]. Surely the origin of this gas is natural (subsoil) but its high concentrations recorded inside the building is also related to the particular microclimatic conditions already abundantly explained. For that reason, the attribution of the input of CO_2_ only to Fiumicino airport is unsure.

Leaving aside the origin of salts, the evaluation of their impact on the materials was also important. To establish a critical level of damage, the obtained concentrations (expressed as a percentage, Table 4) were compared with the maximum levels set by the European standards (Table 5) for each anion.

The results show that all brick samples present a low risk of chlorine contamination, and only some of the samples present a medium risk derived from nitrates, however, this indicates some nitrate input that could likely come also from the acidic gases. Finally, there were some samples with medium and high risk of sulphate contamination, as expected taking in consideration the previous observations.

To solve the restriction of the individual comparisons, the total soluble salt content of the different bricks (taking into consideration both cations and anions) was also compared. The value, around 1.0–5.9% (*w/w),* indicates that the materials were medium/very polluted, which indicates a medium/high risk of hygroscopic moisture and damage to the materials. This fact pointed out the real need to find a solution to the problem of soluble salts to preserve the archaeological site under study.

## 4. Conclusions

The proposed methodology combined with ion chromatography and thermodynamic modelling software (ECOS-RUNSALT and Medusa-Hydra) has allowed us to assess and debate the various salt sources that affected the building, characterized by hygrometric values that exceed 98%. Indeed, it is a very complex scenario due to, not only because the “open museum” is a sensitive place, but also to the multiple environmental stressors that occur in a contemporaneous way.

Notwithstanding the open debate on some questions, it was possible to outline important issues, discretizing some real damage sources. Thanks also to multidisciplinary studies (geophysics, geochemistry, environmental monitoring, and spectroscopic techniques) it was possible to determine and individuate the salt weathering processes and their mechanism of attack, as well as to assess their origin.

One of them regards the soil-salt contamination that involves the first 2.5 m of depth, as below 2.5 m there is a freshwater aquifer linked to the well of the building.

Regarding the possibility of sea spray contamination, it remains an open possibility, even if the salts found and predicted do not mention halite, but only a mixture derived from sulphates. If marine aerosols were attacking the outer walls, according to the thermohygrometric parameters that characterize this building complex, they would remain as uncrystallized salt. It is equally true that the “fractionated infiltration”, or alternative contamination pathways, generates a continuous deposition and removal of salt efflorescence that differs considerably from the composition of sea salt.

The low wind speed (both recorded and simulated) inside the building and near the main openings represents an essential element that allows us to discount long-range pollutant transport. Furthermore, the main wind direction and the km/salinity rate, suggests Fiumicino or the nearby urban city, as the main sources of salt production. Surely, a in depth long-range transport study should be carried out for an ulterior validation, as well a study on the subsoil.

The rising damp (from the freshwater aquifer) also represents a mechanism that should not be underestimated. The hydration represents a fundamental step for the successive salt formation processes. This hydration phenomenon represents the first step of a damage process, making the materials more favorable to salt attack. In any case, the conservation plan to stop the formation of soluble salts should be focused on controlling the RH of the building, that can be reduced by applying small balls of expanded clay or a similar material (changing them at necessary) on the floor.

Despite the uncertainly of the SO_2_ origin, i.e., if it is related to the action of rising damp from the salts circulating present in the soil, or by sea spray (less probable) or both (a synergic action), regarding the CO_2_ input, its origin is clearer. As natural gases are abundantly present in the area surrounding the archaeological site, this had led to the start of a new project to identify and reconstruct the natural gas (CO_2_ and CH_4_) pocket geometry, creating a map of the affected area.

Regarding the CO_2_ levels recorded inside the building they are related to the inadequate air quality (an adequate air movement results in a good air quality) that enables the removal during the day of the CO_2_ emissions caused by biological action (which are added to those produced by high turnover during the day), involving relatively high concentrations of CO_2_ in comparison to the suggested limits and guidelines defined by law.

Finally, the mechanism of the acid attack involves the dry deposition of the atmospheric acid gases.

Regarding the risk level of contamination, most brick samples were medium/very polluted which represents a medium/high risk of hygroscopic moisture and damage to the materials, due to the high presence of sulphate, that attacks indistinctly the red as well as the yellow bricks, without any difference caused by the orientation of the walls. This fact points out a real need to solve the problem of formation of the soluble salts to preserve the studied archaeological site.

## Figures and Tables

**Table 1:** Samples collected (name “S_ID_”, room, and wall orientation). R and Y stands for red and yellow brick, respectively. W_w_ and T_1_ for well water (inside the house) and Tank (outside the house). In the case of T_1_, the sample was collected in a previous study [57].

S_ID_	Room	Orientation
15R	*pre-Mithraeum*	South
15Y		
16R	*Mithraeum*	West
16Y		
17R	*pre-Mithraeum*	West
17Y		
18R	*pre-Mithraeum*	West
18R_2_		West
19R	*Mithraeum*	East
19Y		
20R	*Mithraeum*	North
20Y		
21R	*pre-Mithraeum*	East
21Y		
22R	*Triclinium*	East
22Y		
24S_1_	*Mithraeum*	West
24S_2_	*pre-Mithraeum*	East
W_w_	*Latrinium*	East
T_1_	*Tank*	-

**Table 2:** Chemical composition of sampled bricks (mmol/kg) and water (mmol/L). The bicarbonate value was theoretically determined. The obtained RSD was below 5% in all cases.

S_ID_	Na^+^	K^+^	Mg^2+^	Ca^2+^	F^−^	ClO_2_^−^	Cl^−^	NO_2_^−^	NO_3_^−^	PO_4_^3−^	SO_4_^2−^	HCO_3_^−^
15R	13.1	20.8	18.6	201.4	7.8	0.053	9.0	1.7	5.6	8.1	7.4	410.6
15Y	41.5	20.7	38.7	227.8	7.5	0.045	66.5	1.2	87.6	14.9	8.9	370.0
16R	24.9	27.5	20.3	230.6	10.5	0.053	2.0	1.8	10.4	5.1	37.5	439.3
16Y	31.8	30.3	24.6	273.9	29.1	0.050	3.9	2.7	3.7	6.5	62.2	475.5
17R	79.6	32.1	32.0	444.2	27.8	0.048	13.3	1.0	17.9	26.1	342.3	241.2
17Y	42.3	19.7	26.4	246.1	3.7	0.046	71.6	1.8	35.8	17.5	45.2	351.1
18R	33.0	28.7	19.7	238.0	27.1	0.042	4.9	3.8	6.5	9.7	43.0	419.8
18R_2_	84.6	27.4	42.6	384.1	35.3	0.053	29.5	1.7	38.1	23.8	249.7	200.0
19R	15.0	25.2	11.2	223.6	13.2	0.038	1.0	1.3	<LOD	<LOD	39.6	414.8
19Y	35.0	23.2	34.8	431.0	41.1	0.042	68.0	1.2	21.0	30.3	244.7	278.3
20R	24.9	22.3	20.5	230.7	7.5	0.057	32.9	2.2	18.9	27.4	39.0	328.0
20Y	13.0	24.3	12.1	242.9	18.0	0.042	12.7	1.1	10.5	14.9	13.8	432.6
21R	34.2	26.3	14.6	229.9	10.7	0.050	14.9	1.3	4.2	6.6	19.5	459.6
21Y	19.8	26.5	14.2	194.2	7.6	0.064	3.9	1.7	<LOQ	<LOQ	2.7	445.5
22R	88.9	66.8	38.2	253.5	12.0	0.042	16.4	0.74	9.3	12.6	131.6	399.6
22Y	31.3	18.2	15.1	284.3	5.1	0.042	3.0	1.3	4.9	5.8	125.3	365.7
24S_1_	19.4	14.9	15.1	2831.5	<LOQ	0.327	16.9	<LOD	<LOD	<LOQ	3254.7	-^◊^
24S_2_	55.4	19.4	26.8	1194.9	<LOQ	0.090	7.6b	<LOD	<LOD	<LOQ	924.5	661.4
W_w_	2.7	1.1	0.76	1.1	0.034	n.d.	2.8	n.d.	0.20	0.40	0.33	2.5
* T_1_	2.5	1.0	0.58	0.74	0.022	n.d.	2.1	n.d.	n.d.	n.d.	0.23	3.5

LOD Limit of Quantification (<0.0029 (mmol/L) for all the ions); LOQ Limit of Detection (<0.007 (mmol/L) for all the ions); * T1 [43,44]; ◊ Indicates that cation concentrations are higher compared to anions; n.d. stands for no data.

**Table 3:** Ion correlation analysis for brick samples.

	Na^+^	K^+^	Mg^2+^	Ca^2+^	F^−^	ClO_2_^−^	Cl^−^	NO_2_^−^	NO_3_^−^	PO_4_^3−^	SO_4_^2−^	HCO_3_^−^
**Na^+^**	1											
**K^+^**	0.63	1										
**Mg^2+^**	0.78	0.35	1									
**Ca^2+^**	0.55	0.02	0.59	1								
**F^−^**	0.36	0.11	0.44	0.75	1							
**ClO_2_^−^**	−0.09	−0.13	−0.09	−0.22	−0.18	1						
**Cl^−^**	0.16	−0.24	0.58	0.30	0.07	−0.17	1					
**NO_2_^−^**	−0.27	−0.21	−0.18	−0.21	0.17	0.16	−0.17	1				
**NO_3_^−^**	0.27	−0.22	0.66	0.18	−0.03	−0.11	0.77	−0.15	1			
**PO_4_^3−^**	0.54	−0.06	0.69	0.71	0.54	0.03	0.51	−0.06	0.47	1		
**SO_4_^2−^**	0.70	0.20	0.61	0.96	0.66	−0.18	0.18	−0.27	0.10	0.67	1	
**HCO_3_^−^**	−0.42	0.16	−0.41	−0.58	−0.02	0.10	−0.38	0.39	−0.36	−0.51	−0.66	1

**Table 4:** Summary of the soluble salt results on bricks expressed in weight percentage (*w/w*%). The ionic concentrations considered dangerous by European standards are highlighted, despite other salts being present. The samples that exceed the risk levels are colored in light orange box (low risk), medium orange box (middle risk) and dark orange box (high risk).

S_ID_	Cl^−^	NO_3_^−^	SO_4_^2−^	Total *
15R	0.03%	0.03%	0.07%	**1.2%**
15Y	0.2%	**0.5%**	0.09%	**2.2%**
16R	0.007%	0.06%	0.4%	**1.7%**
16Y	0.01%	0.02%	0.6%	**2.1%**
17R	0.05%	0.1%	**3.3%**	**5.9%**
17Y	0.3%	**0.2%**	0.4%	**2.3%**
18R	0.02%	0.04%	0.4%	**1.8%**
18R_2_	0.1%	**0.2%**	**2.4%**	**5.3%**
19R	0.004%	<LOD	0.4%	**1.5%**
19Y	0.2%	0.1%	**2.4%**	**5.1%**
20R	0.1%	0.1%	0.4%	**2%**
20Y	0.05%	0.07%	0.1%	**1.6%**
21R	0.05%	0.03%	0.2%	**1.5%**
21Y	0.01%	<LOQ	0.03%	**1%**
22R	0.06%	0.06%	**1.3%**	**3.1%**
22Y	0.01%	0.03%	**1.2%**	**2.6%**

* Indicates the total of ionic concentration (chlorides, nitrates and sulfates).

**Table 5:** Degree of contamination by dangerous soluble salts expressed in weight percentage (*w/w*%) according to WTA (International Association for Science and Technology of Building Maintenance and the Preservation of Monuments).

Risk	[Cl^−^]	[NO_3_^−^]	[SO_4_^2−^]	Total
Low	<0.3%	<0.1	<0.8%	<1.2%
Middle	0.3–0.8%	0.1–0.5%	0.8–1.6%	1.2–2.9%
High	>0.8%	>0.5%	>1.6%	>2.9%

## Data Availability

The datasets generated and analysed during the current study are available from the corresponding author on reasonable request.

## Sample Availability

Samples are not available from the authors.
